# Immune Checkpoint Inhibitors With or Without Bone-Targeted Therapy in NSCLC Patients With Bone Metastases and Prognostic Significance of Neutrophil-to-Lymphocyte Ratio

**DOI:** 10.3389/fimmu.2021.697298

**Published:** 2021-11-10

**Authors:** Alberto Bongiovanni, Flavia Foca, Jessica Menis, Stefania Luigia Stucci, Fabrizio Artioli, Valentina Guadalupi, Maria Rosachiara Forcignanò, Manuela Fantini, Federica Recine, Laura Mercatali, Chiara Spadazzi, Marco Angelo Burgio, Valentina Fausti, Anna Miserocchi, Toni Ibrahim

**Affiliations:** ^1^ Osteoncology and Rare Tumors Center (CDO-TR), IRCCS Istituto Romagnolo per lo Studio dei Tumori (IRST) “Dino Amadori”, Meldola, Italy; ^2^ Unit of Biostatistics and Clinical Trials, IRCCS Istituto Romagnolo per lo Studio dei Tumori (IRST) “Dino Amadori”, Meldola, Italy; ^3^ Department of Surgery, Oncology and Gastroenterology, University of Padova, Padova, Italy; ^4^ Medical Oncology Department, Istituto Oncologico Veneto IRCCS, Padova, Italy; ^5^ Medical Oncology, Department of Medicine, University of Verona, Azienda Ospedaliera Universitaria Integrata (AOUI) di Verona, Verona, Italy; ^6^ Medical Oncology Unit, Policlinico Hospital of Bari Department of Biomedical Sciences and Human Oncology University of Bari “A. Moro”, Bari, Italy; ^7^ Division of Medical Oncology, Ramazzini Hospital, Carpi, Italy; ^8^ IRCCS National Cancer Institute (INT), Milan, Italy; ^9^ UOC di Oncologia Medica Ospedale Sacro cuore di Gesù, Gallipoli, Italy; ^10^ Oncology Unit, Infermi Hospital, Rimini, Italy; ^11^ San Camillo De Lellis Hospital, ASL Rieti, Rieti, Italy; ^12^ Department of Medical Oncology, IRCCS Istituto Romagnolo per lo Studio dei Tumori (IRST) “Dino Amadori”, Meldola, Italy

**Keywords:** immune checkpoint inhibitors, NSCLC, bone metastases, zoledronate, denosumab, lung cancer

## Abstract

**Introduction:**

Bone metastases (BMs) are a negative prognostic factor in patients with non-small cell lung cancer (NSCLC). Although immune-checkpoint inhibitors (ICIs) have dramatically changed the therapeutic landscape of NSCLC, little information is available on BMs from NSCLC treated with ICIs alone or in association with bone-targeted therapy (BTT) such as zoledronate or denosumab.

**Methods:**

From 2014 to 2020, 111 of the 142 patients with BMs secondary to NSCLC extrapolated from the prospective multicenter Italian BM Database were eligible for analysis. Information on blood count, comorbidities, and toxicity was retrospectively collected. The neutrophil-to-lymphocyte ratio (NLR) pre- and post-treatment was calculated. Survival was analyzed using the Kaplan–Meier method, with statistical significance of survival differences assessed using the log-rank test.

**Results:**

Median age was 66 (range, 42–84) years. Performance status (PS) Eastern Cooperative Oncology Group (ECOG) was 0–1 in 79/111 patients. The majority of patients (89.2%) had adenocarcinoma histology. At a median follow-up of 47.4 months, median progression-free (mPFS) and overall survival (mOS) was 4.9 (95%CI, 2.8–10.0) and 11.9 (95%CI, 8.2–14.4) months, respectively. Forty-six (43.4%) patients with BM NSCLC underwent first- or further-line therapy with ICIs: 28 (60.8%) received nivolumab, 9 (19.6%) pembrolizumab, and 9 (19.6%) atezolizumab. Of the 46 patients treated with ICIs, 30 (65.2%) underwent BTT: 24 (80.0%) with zoledronate and 6 (20.0%) with denosumab. The ICI-alone group had an mOS of 15.8 months [95%CI, 8.2–not evaluable (NE)] *vs*. 21.8 months (95%CI, 14.5–not evaluable) for the ICI plus BTT group and 7.5 (95%CI, 6.1–10.9) months for the group receiving other treatments (p < 0.001). NLR ≤5 had a positive impact on OS.

**Conclusion:**

BTT appears to have a synergistic effect when used in combination with ICIs, improving patient survival.

## Introduction

Lung cancer remains, independently of gender, one of the leading causes of cancer death worldwide (1). Despite the therapeutic breakthrough following the development of molecular-targeted therapies and immune checkpoint inhibitors (ICIs), the prognosis of patients with metastatic disease, albeit highly variable, remains poor (2). Previous studies and routine clinical practice have confirmed that ICIs show good tolerance and clinical efficacy in patients with advanced or recurrent non-small cell lung cancer (NSCLC) in both first- and further-line settings (3–9). Nevertheless, the effect on specific subgroups warrants further investigation.

The bone is one of the most common sites of metastasis in NSCLC, with 30–66% of patients developing bone metastases (BMs) during the course of their disease (10). BMs usually appear as mainly lytic, mainly osteoblastic, or mixed lesions and are excluded from Response Evaluation Criteria in Solid Tumors (RECIST) because they are difficult to measure (11). For this reason, in 2004, Hamaoka et al. developed the MD Anderson response classification criteria (MDA criteria), which are specific for the assessment of BMs (12, 13).

Recently, a negative effect of BMs was seen in large populations of NSCLC patients treated with nivolumab, independently of the presence of brain or liver metastases or of poor Performance Status (PS) (14, 15). One explanation for this may be related to the immunosuppressive status of the tumor microenvironment (TME), which, in some patients, cannot be effectively reversed after ICI therapy (16).

In a preclinical breast cancer mouse model, a combination of anti-PD-1 antibody plus zoledronic acid induced a better antitumor response than untreated controls or single-agent treatment, without significant toxicity (17). The RANKL/RANK signaling pathway also appears to modulate the immune microenvironment and enhance the efficacy of anti-CTLA-4 and anti-PD-1 monoclonal antibodies against solid tumors. This positive synergistic effect has also been suggested in real-world studies on patients with metastatic melanoma and NSCLC (18, 19).

In addition to PD‐L1 expression, tumor mutational burden, and mismatch repair deficiency or microsatellite instability, several other potential biomarkers have been investigated or are currently under evaluation (20). Despite the large-scale use of immunotherapy in early and advanced NSCLC, there are still no validated or reliable predictive biomarkers of response or resistance to immunological agents (2).

Although some authors have reported a predictive and prognostic role of the neutrophil-to-lymphocyte ratio (NLR) in patients with advanced NSCLC undergoing different systemic treatments, its role in NCSLC patients with BMs has yet to be clarified (21–26).

Given the above premises, we decided to investigate the efficacy and safety of ICIs in NSCLC patients with BMs treated with zoledronate or denosumab, usually referred to as bone-targeted therapy (BTT). We also explored the relationship between bone response and tumor control in patients treated with ICIs and evaluated the potential predictive and prognostic role of NLR in this population.

## Material and Methods

### Study Design, Patients, and Treatment

The present analysis was performed on information extrapolated from our Bone Metastasis Database (BMDB) and from retrospectively collected data. The Italian BMDB was a prospective, observational multicenter project designed to collect data on BMs from solid tumors. Details on the project and its main inclusion and exclusion criteria have been described elsewhere (27).

Briefly, we extrapolated data on patients aged ≥18 years, with a histological or cytological diagnosis of NSCLC, treated for advanced disease, and with Eastern Cooperative Oncology Group (ECOG) Performance Status (PS). Patients included in the analysis had received at least one dose of ICIs as first- or further-line treatment. Blood count, comorbidities, presence of brain metastases, and safety information were retrospectively collected. NLR was calculated by dividing neutrophils and lymphocytes measured in peripheral blood. We recorded the NLR before ICI +/− BTT treatment and at response. This study was conducted in accordance with the International Conference of Harmonization Guidelines for Clinical Practice and the principles laid down in the 1964 Declaration of Helsinki. The protocol was approved by the Institutional Review Board of each participating center. All patients provided written informed consent.

### Outcome Measures

Tumor response was assessed using RECIST criteria version 1.1 (28). Investigator-assessed progression-free survival (PFS) and overall survival (OS) were evaluated. OS was calculated for all patients as the time between the fist diagnosis of BM and the date of death or date of last follow-up visit. PFS was calculated for the subgroup of patients undergoing ICI +/− BTT as the time from the start of treatment until the first documented evidence of progressive disease (PD) or death, whichever occurred first. Patients were monitored for adverse events (AEs) using the National Cancer Institute Common Terminology Criteria for Adverse Events v4.0. MDA criteria were used to evaluate bone response (12, 29). A multidisciplinary group dedicated to bone evaluation was involved to better clarify the bone response (30).

### Statistical Analysis

Objective response rate (ORR), PFS, OS, and safety were assessed. Reverse Kaplan–Meier method was used to estimate median follow-up. Efficacy and safety analyses were conducted on all patients who received at least one ICI dose. The chi-square test was used to evaluate the association between patient characteristics and ORR. PFS and OS were estimated using the Kaplan–Meier method, and 95% confidence intervals (95%CIs) were reported. Differences between survival curves were evaluated with the log-rank test. The Wilcoxon signed-rank test was used to compare pre- and post-treatment NLR values.

## Results

### Patient Characteristics

**Figure 1:**
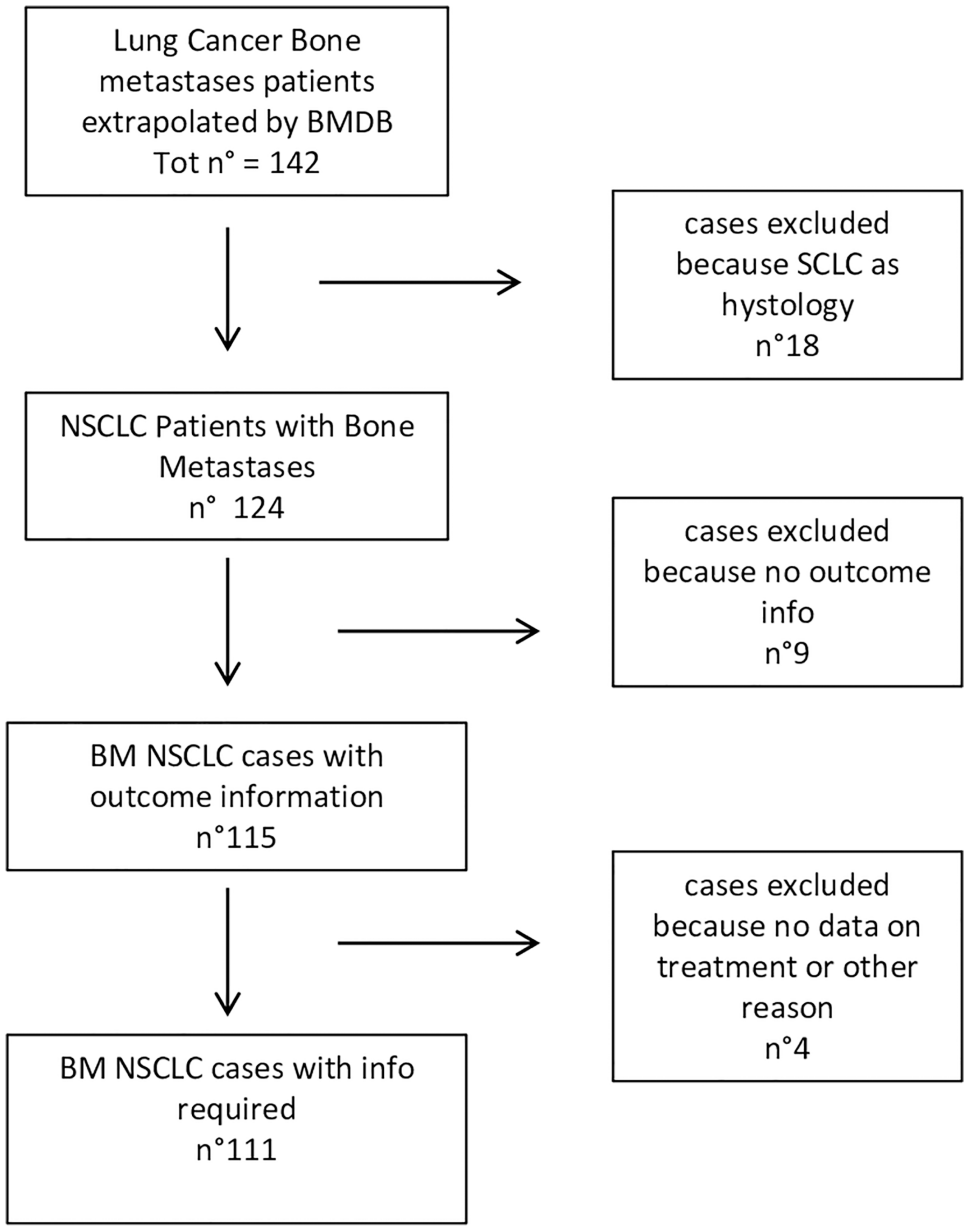
Flow chart of patient selection.

From January 2014 to December 2020, 142 patients with BMs were selected from the lung cancer cohort of our BMDB, and 111 (78.2%) were eligible for analysis. Median age was 66 years (range, 42–84). Forty-six (43.4%) of the 111 patients had been treated with an ICI, 28 (60.8%) with nivolumab, 9 (19.6%) with pembrolizumab, and 9 (19.6%) with atezolizumab (**Figure 1**). Thirty-five patients only had one comorbidity (cardiovascular), while 44 patients had more than one comorbidity (**Table 1** and **Supplementary Table S1**).

**Table 1:** Demographics and clinical characteristics of NSCLC patients (n = 111).

	Patients (n = 111)
**Median age, years, at diagnosis of first bone metastasis (range)**	**66 (42–84)**
	**No. (%)**
**Age (years) at diagnosis of primary bone metastasis**	
≤65	47 (42.3)
>65	64 (57.7)
**Gender**	
Male	70 (63.1)
Female	41 (36.9)
**ECOG PS at diagnosis of first bone metastasis**	
0	26 (30.6)
1	53 (62.4)
≥2	6 (7.0)
Unknown	26
**Histology**	
Adenocarcinoma	99 (89.2)
Squamous carcinoma	9 (8.1)
Large-cell carcinoma	1 (0.9)
Adenosquamous carcinoma	1 (0.9)
Undifferentiated	1 (0.9)
**Grading (G)**	
1	2 (4.1)
2	13 (26.5)
3	32 (65.3)
4	2 (4.1)
Unknown	62
**Presence of visceral metastasis**	
Yes	91 (82.0)
No	20 (18.0)
**Presence of brain metastasis**	
Yes	18 (22.0)
No	64 (78.0)
Unknown	29
**Presence of comorbidity**	
Cardiovascular	35 (42.2)
Cardiovascular + other^*^	10 (12.0)
Other^§^	5 (6.0)
None	33 (39.8)
Unknown	28

^*^Cardiovascular + other: 4 cardiovascular + diabetes; 1 cardiovascular + diabetes + renal impairment + other; 1 cardcardiovascular + diabetes + other; 2 cardiovascular + renal impairment; 2 cardiovascular + other.

^§^Other: 2 diabetes, 1 renal impairment; 1 other, 1 diabetes + renal impairment.

Twenty (18.0%) patients only had BMs. Of those with both BMs and visceral metastases (n = 91, 81.9%), 18 (22.0%) also had brain lesions. The majority of patients had mainly osteolytic BMs (n = 77, 77.0%), 6 (6.0%) had mainly osteoblastic BMs, and 17 (17.0%) had mixed BMs. This information was not available for 11 patients. Fifty-four (48.7%) patients had multiple (>6) bone metastases, 33 (29.7%) had two to six BMs, and 24 (21.6%) had only one BM. Only five patients (11.6%) received bone radiotherapy (**Supplementary Table S2**).

We also recorded information on the molecular profile of tumors. Epidermal growth factor receptor (EGFR) mutation was present in 12 (12.5%) cases and wild type in 84 (87.5%). c-Ros oncogene 1 (ROS1) was rearranged in 3 (5.9%) patients and wild type in 48 (94.1%). Anaplastic lymphoma kinase (ALK) translocation was detected in 4 (5.6%) of the 67 patients in which it was evaluated. Ten (28.6%) of the 35 patients analyzed for KRAS (Kirsten rat sarcoma viral oncogene homolog) showed a mutation (**Table 2**).

**Table 2:** Biological characteristics of NSCLC patients (n = 111).

	No. (%)
**EGFR**	
Mutated	12 (12.5)
Wild type	84 (87.5)
Not evaluated	12
Unknown	3
**ALK**	
Translocated	4 (5.6)
Wild type	67 (94.4)
Not evaluated	26
Unknown	14
**ROS1**	
Rearranged	3 (5.9)
Wild type	48 (94.1)
Not evaluated	30
Unknown	30
**KRAS**	
Mutated	10 (28.6)
Wild type	25 (71.4)
Not evaluated	48
Unknown	28
**PDL1 (1)**	
<50%	29 (76.3)
≥50%	9 (23.7)
Unknown	73
**PDL1 (2)**	
<1%	14 (36.8)
≥1%	24 (63.2)
Unknown	73

EGFR, epidermal growth factor receptor; ALK, anaplastic lymphoma kinase; ROS1, c-ros oncogene 1; KRAS, Kirsten rat sarcoma viral oncogene homolog; PDL1, programmed death-ligand 1.

### Patient Outcome

At a median follow-up of 41.4 months, the median OS (mOS) of the entire population was 11.9 months (95%CI, 8.2–14.4). Of the 46 patient treated with ICIs, 30 (65.2%) underwent BTT, 24 (80.0%) with zoledronate, and 6 (20.0%) with denosumab. In all patients treated with ICI +/− BTT, the median PFS (mPFS) and mOS were 4.9 (95%CI, 2.8–10.0) and 19.2 (95%CI, 13.6–36.8) months, respectively.

No differences were seen according to the RECIST 1.1 criteria response in the two groups (**Supplementary Table S2**).

With regard to bone response evaluated using MD Anderson criteria, 10 (43.5%) patients obtained a partial response (PR) following ICIs plus BTT, while only 2 (16.7%) obtained the same response when treated with ICIs alone. In the latter group, stable disease (SD) as a response was more frequent than in the combination group (*p* = 0.042) (**Supplementary Table S3**).

**Figure 2:**
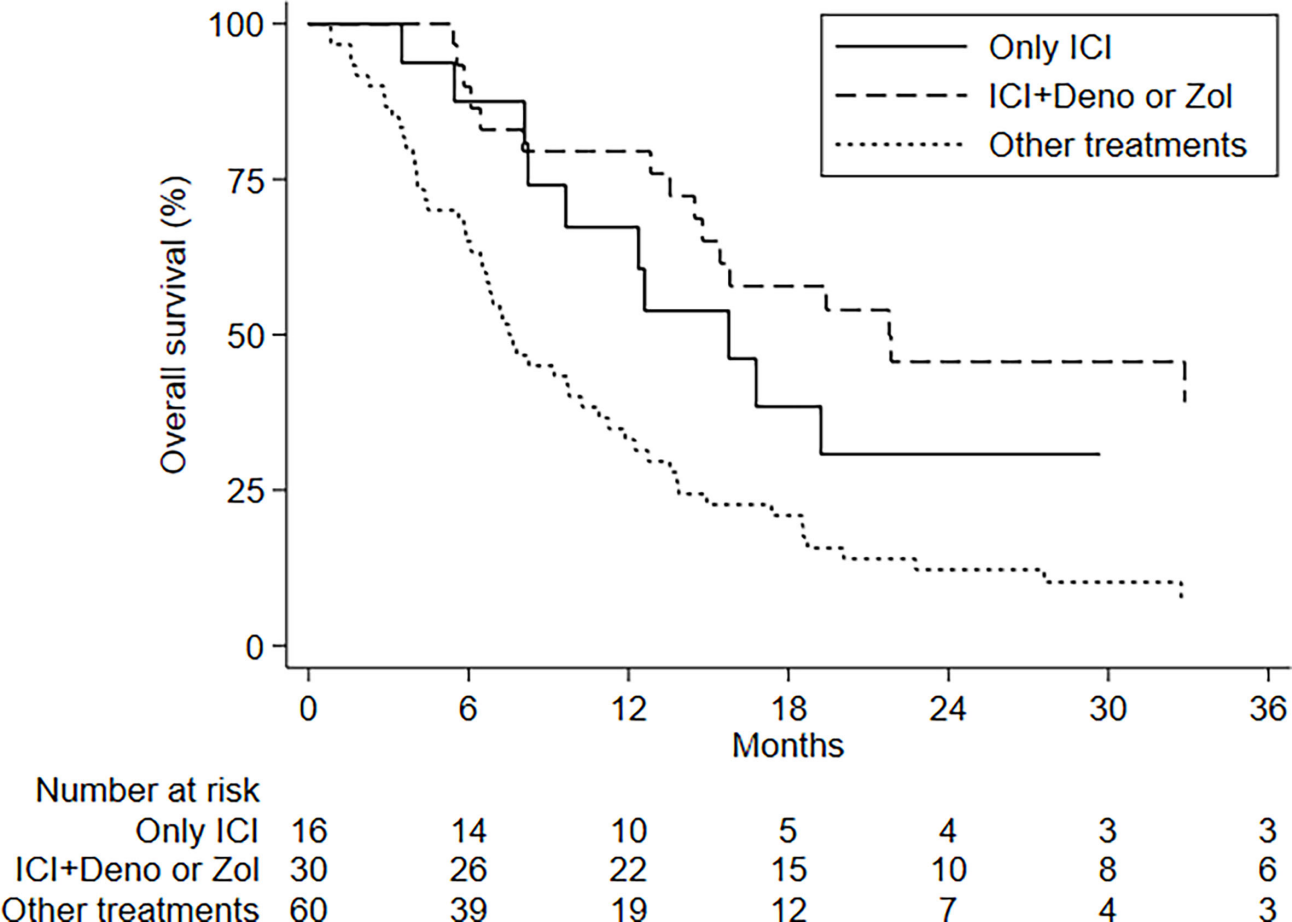
OS by treatment.

Patients treated with ICIs plus BTT had an mOS of 21.8 months (95%CI, 14.5–NE) and a 24-month OS rate of 45.7% (95%CI, 26.5–62.9); those undergoing ICIs alone showed an mOS of 15.8 months (95%CI, 8.2–NE) and a 24-month OS of 30.8% (95%CI, 9.9–54.8); and the group receiving other treatments had an mOS of 7.5 months (95%CI, 6.1–10.9) and a 24-month OS of 12.2% (95%CI, 5.4–21.9). This difference was statistically significant (*p* < 0.001) (**Figure 2**).

There was no difference in PFS between patients treated or not with BTT, although those receiving denosumab (n = 6) had a better mPFS (15.9 months; 95%CI, 5.1–not estimable) than patients treated with ICIs alone or with zoledronate (*p* = 0.068).

### Prognostic and Predictive Factors Evaluation

**Figure 3:**
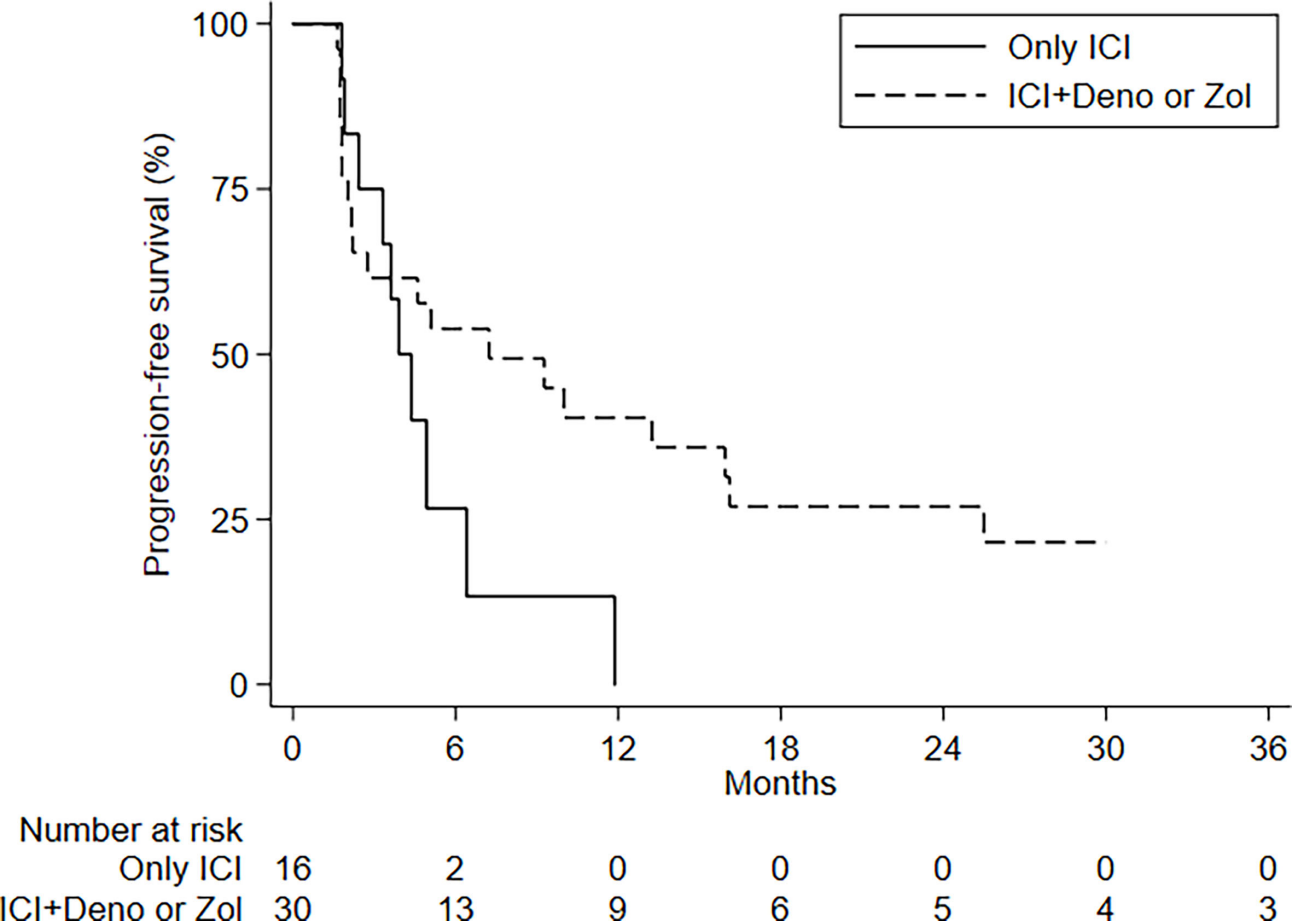
PFS by treatment.

**Figure 4:**
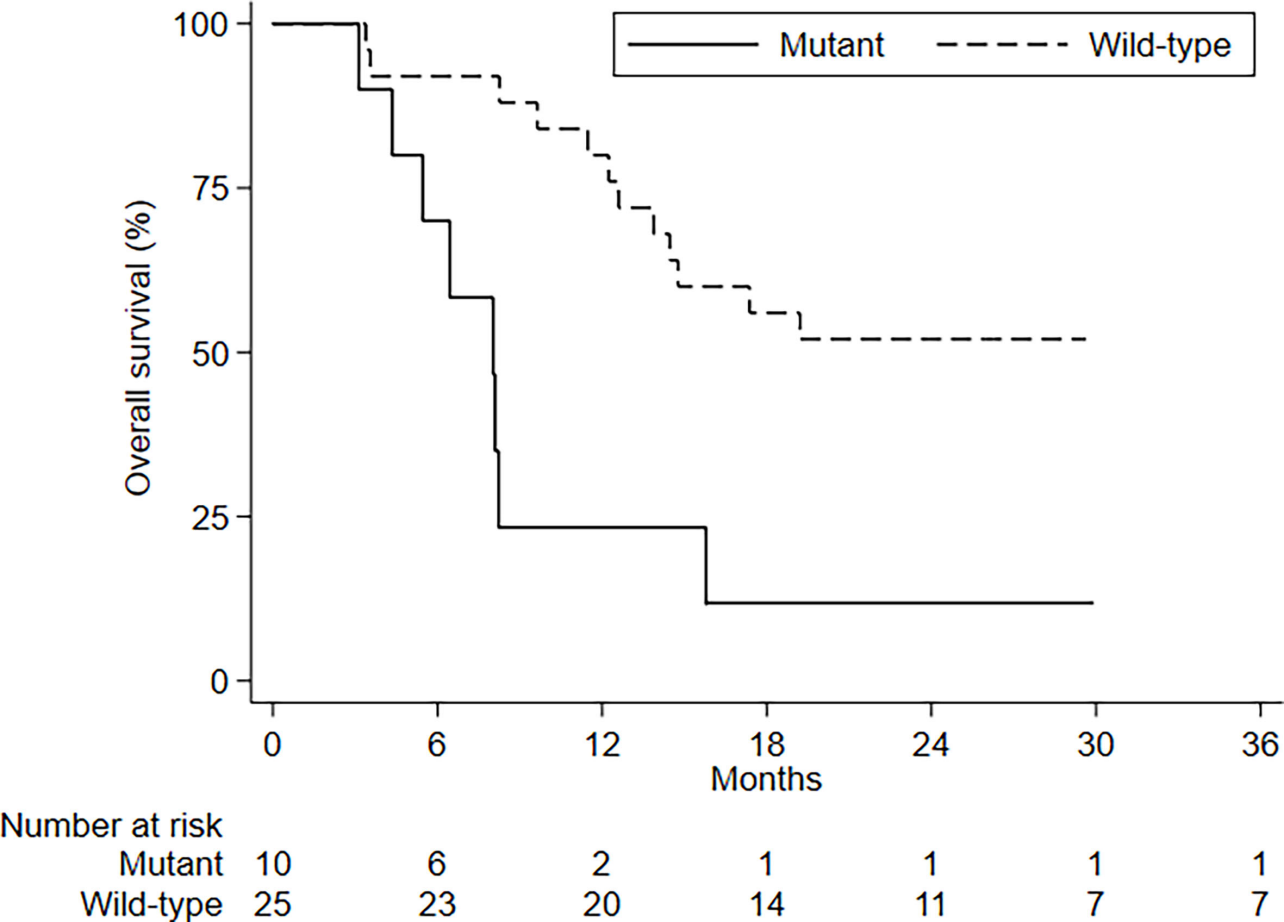
OS by KRAS status.

There were no differences in PFS and OS in relation to the number of BMs, type of BM, presence of visceral metastases, and age (**Figure 3**). ECOG PS had an impact on OS but not on PFS (**Supplementary Table S5**). No differences in PFS and OS were seen in relation to PDL1 status and tumor molecular profile, with the exception of KRAS mutations; patients with KRAS-mutated disease had an mOS of 8 months (95%CI, 4.3–8.2–NE) compared to 38.8 months (95%CI, 13.9–NE) for those with KRAS wild-type tumors (**Figure 4** and **Tables 3**, **4**).

**Table 3:** Univariable analysis of overall survival.

	No. patients	No. events	Median OS(95%CI)	12-month OS(95%CI)	24-month OS (95%CI)	*p*-valuelog-rank test
**Overall no. patients**	111	88	11.9 (8.2–14.4)	49.7 (40.0–58.7)	24.0 (16.3–32.6)	–
**Age, years, at first diagnosis of BM**						
<65	47	36	13.9 (8.0–18.6)	56.4 (40.8–69.3)	29.1 (16.7–42.8)	0.235
≥65	64	52	10.3 (6.9–13.6)	45.0 (32.5–56.7)	20.4 (11.2–31.6)	
**Gender**						
Male	70	54	12.4 (8.0–15.4)	50.9 (38.5–61.9)	21.6 (12.6–32.2)	0.842
Female	41	34	11.9 (5.8–15.8)	47.7 (31.7–62.1)	28.4 (15.2–43.1)	
**ECOG at first diagnosis of BM**						
0	26	14	32.9 (12.8–NE)	76.9 (55.7–88.9)	57.2 (36.1–73.6)	0.002
1	53	42	12.6 (7.2–15.4)	53.6 (39.1–66.0)	20.6 (10.4–33.1)	
≥2	6	6	5.6 (3.9–NE)	16.7 (0.7–51.7)	0	
**Treatment**						
ICI alone	16	11	15.8 (8.2–NE)	63.0 (38.3–84.9)	30.8 (9.9–54.8)	<0.001
ICI+Deno/Zol	30	17	21.8 (14.5–NE)	79.5 (59.9–90.2)	45.7 (26.5–62.9)	
No ICI	60	55	7.5 (6.1–10.9)	33.1 (21.5–45.1)	12.2 (5.4–21.9)	
**Visceral metastasis**						
No	20	16	14.9 (5.8–27.6)	53.6 (29.6–72.6)	37.5 (16.9–58.2)	0.413
Yes	91	72	11.9 (8.2–13.8)	48.9 (38.1–58.7)	20.9 (13.0–30.2)	
**No. BMs**						
1	24	19	15.4 (7.5–19.4)	59.9 (36.9–76.8)	23.0 (8.4–41.8)	0.761
2–6	33	26	9.8 (6.5–13.8)	48.5 (30.8–64.0)	25.9 (12.4–41.7)	
>6	54	43	10.9 (6.7–15.8)	46.1 (32.4–58.7)	23.1 (12.5–35.6)	
**Type of bone lesion**						
Osteoblastic	6	6	14.9 (1.6–NE)	66.7 (19.5–90.4)	–	0.386
Lytic	77	57	11.5 (8.2–15.8)	49.6 (37.8–60.3)	29.6 (19.6–40.3)	
Mixed	17	15	9.8 (5.4–15.4)	47.1 (22.9–67.9)	17.7 (4.3–38.3)	
**EGFR status**						
Mutated	12	10	12.2 (5.6–NE)	58.3 (27.0–80.1)	25.0 (6.0–50.5)	0.937
Wild type	84	65	12.6 (8.0–14.9)	50.3 (39.0–60.5)	25.4 (16.3–35.4)	
**ALK status**						
Translocated	4	2	–	–	–	–
Wild type	67	52	12.7 (8.2–14.9)	54.3 (41.5–65.4)	26.5 (16.3–37.9)	
**ROS1 status**						
Rearranged	3	2	–	–	–	
Wild type	48	32	13.9 (11.5–22.8)	63.6 (48.1–75.6)	35.8 (22.1–49.8)	
**KRAS status**						
Mutant	10	8	8.0 (3.1–15.8)	23.3 (3.6–52.9)	11.7 (0.6–40.1)	0.005
Wild type	25	15	36.8 (13.9–NE)	80.0 (58.4–91.2)	52.0 (31.3–69.2)	
**PDL1 (1)**						
<50%	29	19	15.7 (12.6–48.6)	78.8 (58.7–89.8)	37.7 (19.9–55.4)	0.995
≥50%	9	4	13.9 (5.4–NE)	63.5 (23.8–86.6)	47.6 (12.3–76.9)	
**PDL1 (2)**						
<1%	14	8	15.8 (13.6–NE)	100.0	42.8 (17.7–66.0)	0.275
≥1%	24	15	12.8 (8.2–NE)	59.4 (36.3–76.5)	38.2 (17.7–58.5)	
**Mutational status (1)**						
EGFR mutated	12	10	12.2 (5.6–NE)	58.3 (27.0–80.0)	25.0 (6.0–50.5)	0.114
ALK translocated	4	2	–			
KRAS mutated	10	8	8.0 (4.3–15.8)	23.3 (3.6–52.9)	11.7 (0.6–40.0)	
ROS1 rearranged	3	2	–			
EGFR, ALK, KRAS and ROS1 wild type	14	10	14.7 (9.6–NE)	71.4 (40.6–88.2)	42.9 (17.7–66.0)	
**Mutational status (2)**						
EGFR mutated or ALK translocated or ROS1 rearranged	19	14	17.4 (9.7–48.6)	73.7 (47.9–88.1)	36.8 (16.5–57.5)	0.778
None	36	24	14.5 (9.6–27.2)	62.5 (44.2–76.3)	37.3 (21.1–53.4)	

PFS, progression-free survival; 95%CI, 95% confidence interval; ECOG PS, Eastern Cooperative Oncology Group Performance Status; BM, bone metastasis; ICI, immune checkpoint inhibitor; Deno, denosumab; Zol, zoledronate; NE, not estimable; EGFR, epidermal growth factor receptor; ALK, anaplastic lymphoma kinase; ROS1, c-ros oncogene 1; KRAS, Kirsten rat sarcoma viral oncogene homolog; PDL1, programmed death-ligand 1.

**Table 4:** Univariable analysis of progression-free survival.

	No. patients	No. events	Median PFS (95%CI)	6-month PFS (95%CI)	12-month PFS (95%CI)	*p*-valuelog-rank test
**Overall no. patients**	46	30	4.9 (2.8–10.0)	47.4 (29.9–61.4)	29.9 (15.5–45.6)	–
**Age, years, at first diagnosis of BM**						
<65	18	11	6.4 (2.0–11.9)	57.1 (28.4–77.9)	11.4 (0.7–39.0)	0.698
≥65	28	19	4.6 (2.2–15.9)	40.6 (21.1–59.3)	36.1 (17.6–55.0)	
**Gender**						
Male	35	23	4.9 (2.2–10.0)	47.3 (28.3–64.2)	25.8 (10.9–34.7)	0.451
Female	11	7	5.1 (2.0–38.1)	44.4 (13.6–71.9)	44.4 (13.6–71.9)	
**ECOG PS at first diagnosis of BM**						
0	15	9	7.2 (2.1–NE)	54.5 (22.9–77.9)	21.8 (3.5–50.1)	0.955
1	29	21	4.9 (1.9–13.3)	43.5 (24.5–61.1)	33.8 (16.3–52.3)	
≥2	2	0	–	–	–	
**Treatment**						
Only ICIs	16	10	3.9 (1.9–6.4)	26.7 (5.1–55.6)	–	0.068
ICI+Deno	6	4	15.9 (5.1–NE)	83.3 (27.3–97.5)	66.7 (19.5–90.4)	
ICI+Zol	24	16	2.7 (1.8–13.3)	45.0 (23.1–65.7)	32.1 (12.8–53.4)	
**Presence of visceral metastasis**						
No	7	2	–	–	–	–
Yes	39	28	4.6 (2.2–9.3)	43.6 (26.7–59.3)	29.1 (14.4–45.5)	
**No. BMs**						
1	11	8	4.3 (1.7–16.1)	50.0 (18.4–75.3)	25.0 (4.1–54.9)	0.343
2–6	15	8	4.9 (2.4–NE)	45.5 (16.7–70.7)	36.4 (11.2–62.7)	
>6	20	14	5.1 (2.2–13.3)	45.4 (20.9–67.2)	27.2 (7.4–52.1)	
**Type of bone lesion**						
Osteoblastic	1	0	–	–	–	–
Lytic	38	24	6.4 (3.6–11.9)	51.9 (33.3–67.7)	31.9 (15.8–49.4)	
Mixed	6	5	1.8 (1.7–NE)	20.0 (0.8–58.2)	20.0 (0.8–58.2)	
Unknown	1	1	–	–	–	
**EGFR status**						
Mutant	1	0	–	–	–	–
Wild–type	38	24	5.1 (2.2–11.9)	47.8 (29.5–64.0)	31.9 (15.8–49.2)	
**ALK status**						
Translocated	0	–	–	–	–	–
Wild type	35	22	4.9 (2.4–9.3)	44.7 (26.5–62.2)	29.8 (13.1–48.6)	
**ROS1 status**						
Rearranged	1	–	–	–	–	–
Wild–type	30	20	5.1 (2.7–13.3)	48.1 (27.7–65.9)	32.1 (14.1–51.7)	
**KRAS status**						
Mutant	8	3	4.9 (1.7–NE)	44.4 (6.6–78.5)	44.4 (6.6–78.5)	0.565
Wild-type	13	11	7.2 (2.4–11.9)	61.5 (30.8–81.8)	20.5 (33.3–47.8)	
**PDL1 (1)**						
<50%	23	15	4.4 (2.2–7.2)	37.1 (16.3–58.2)	14.9 (2.6–36.8)	0.810
≥50%	7	6	5.1 (1.7–NE)	42.8 (9.8–73.4)	42.8 (9.8–73.4)	
**PDL1 (2)**						
<1%	12	8	4.6 (1.6–9.3)	50.0 (18.4–75.3)	12.5 (0.7–41.8)	0.941
≥1%	18	13	4.3 (2.2–13.3)	30.9 (10.5–54.3)	30.9 (10.5–54.3)	

PFS, progression-free survival; 95%CI, 95% confidence interval; ECOG PS, Eastern Cooperative Oncology Group Performance Status; BM, bone metastasis; ICI, immune checkpoint inhibitor; Deno, denosumab; Zol, zoledronate; NE, not estimable; EGFR, epidermal growth factor receptor; ALK, anaplastic lymphoma kinase; ROS1, c-ros oncogene 1; KRAS, Kirsten rat sarcoma viral oncogene homolog; PDL1, programmed death-ligand 1.

**Figure 5:**
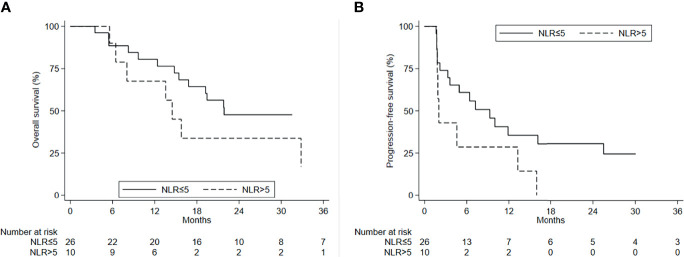
**(A)** OS and **(B)** PFS by NLR values.

The mean NLR value in patients treated with ICI +/− BTT was 4.08 [standard deviation (SD), 1.83]. A statistically significant difference in OS was observed according to the basal value of NLR (**Figure 5A**). In particular, patients treated with ICIs alone or ICIs + BTT and with an NLR ≤5 had a better mOS (21.8 months; 95%CI, 15.4–NE) than those with an NLR >5 (14.5 months; 95%CI, 5.6–32.9). This difference was significant (*p* = 0.042) (**Supplementary Table S6**). There was also a positive trend for PFS, with an mPFS of 9.3 months (95%CI, 3.3–25.4) in the former group and 2.0 months (95%CI, 1.7–13.2) in the latter group (**Figure 5B**) (*p* = 0.086). However, patients who obtained PR or SD on ICIs +/− BTT showed a decrease in NLR with respect to NLR at best response [basal NLR value, 3.52 (SD, 1.56) *vs*. best response, 2.78 (SD, 1.64)] (*p* = 0.030) (**Supplementary Figure S1**). Conversely, NLR increased in patients progressing after ICIs +/− BTT [mean basal NLR value, 3.65 (SD, 1.42) *vs*. 5.18 at progression (SD, 2.79)] (*p* = 0.027).

### Safety

Patients treated with ICIs had mild and reversible toxicities (**Table 5**). In the combination group, six cases of grade (G)1 hypocalcemia, three cases of G1 renal toxicity, and one case of osteonecrosis of the jaw were reported. One case of G2 renal creatinine increase was recorded in the ICI-alone group. There were few cases of G3 toxicities (arthralgia, increased amylase and lipase, and dermatitis) related to ICI therapy, all of which were successfully resolved. The safety profile was consistent with literature data.

**Table 5:** Toxicities recorded in both ICI and ICI+BTT treatments.

Toxicity	Grade			Total No. (%)
	1No. (%)	2No. (%)	3No. (%)	
Arthralgia	1 (2.2)	0 (0.0)	1 (2.2)	2 (4.3)
Asthenia	2 (4.3)	0 (0.0)	1 (2.2)	3 (6.5)
Dermatitis	0 (0.0)	0 (0.0)	1 (2.2)	1 (2.2)
Diarrhea	2 (4.3)	1 (2.2)	0 (0.0)	3 (6.5)
Dypnea	0 (0.0)	1 (2.2)	0 (0.0)	1 (2.2)
Infection	0 (0.0)	1 (2.2)	0 (0.0)	1 (2.2)
Creatinine increase	5 (10.9)	1 (2.2)	0 (0.0)	6 (13.0)
Hyperamylasemia	1 (2.2)	0 (0.0)	1 (2.2)	2 (4.3)
Hypertransaminasemia	2 (4.3)	0 (0.0)	1 (2.2)	3 (6.5)
Hypophosphatemia	0 (0.0)	1 (2.2)	0 (0.0)	1 (2.2)
Hypothyroidism	1 (2.2)	0 (0.0)	0 (0.0)	1 (2.2)
Neuropathy	0 (0.0)	1 (2.2)	0 (0.0)	1 (2.2)
Neutropenia	0 (0.0)	1 (2.2)	0 (0.0)	1 (2.2)
Pneumonitis	0 (0.0)	2 (4.3)	0 (0.0)	2 (4.3)
Skin rash	1 (2.2)	1 (2.2)	0 (0.0)	2 (4.3)
Lipase increase	1 (2.2)	0 (0.0)	1 (2.2)	2 (4.3)
Sepsis	0 (0.0)	0 (0.0)	1 (2.2)	1 (2.2)
Skin toxicity	0 (0.0)	0 (0.0)	1 (2.2)	1 (2.2)

## Discussion

ICIs have dramatically changed the treatment of patients with NSCLC (2). However their immune-mediated antitumor activity is dependent on several complex mechanisms, also involving the microenvironment. In BMs, the microenvironment is represented by a particular landscape characterized by reciprocal interactions between cancer cells, local stromal cells, immune cells, and several other factors such as osteoclasts (members of the mononuclear-macrophage family) and cytokines (31).

The results from two large phase III studies, CheckMate 227 and CheckMate 057, not only suggested that bone involvement may be a negative prognostic factor in patients with metastatic NSCLC, but also that the presence of BMs could be predictive of a poor response to ICIs (32, 33). However, none of the randomized trials on immunotherapy, including CheckMate 227, stratified patients on the basis of the site of metastasis, thus precluding any definitive conclusions from being drawn (34).

In our study, the poor outcome of NSCLC patients with bone metastases was confirmed in patients treated or not with ICIs, the latter showing an mOS of 7.8 months.

This interest in defining the role of immunotherapy on the basis of the site of metastasis and, in particular, the bone (10–12) prompted us to explore this area using data extrapolated from the Italian BMDB. A strong point in our favor is that the characteristics, outcome, and safety data of the patients who received ICIs are consistent with literature data (35), thanks to the multicenter nature of our BMDB, the largest of its kind in Italy.

A recent study stressed the concept of the negative modulation of the immune response by BMs in NSCLC (15). However, data on the concomitant use of BTTs were not collected. The hypothesis of the potential immunomodulatory effect of BTTs such as denosumab and zoledronate has been gaining ground worldwide over the past two decades.

There is evidence from preclinical research into prostate cancer and breast cancer mouse models of the immunomodulatory effect of zoledronate and of its enhancement of the antitumor efficacy of the PD-1 blockade (17). Nitrogen-containing bisphosphonates (N-BPs) such as zoledronate inhibit farnesyl pyrophosphate synthesis in the mevalonate pathway, leading to increased levels of isopentenyl pyrophosphate in tumor cells, which renders them targets of Vγ9Vδ2 T cells, and thus contributing to innate immunity (36).

Although denosumab added to chemotherapy did not modify OS with respect to CT alone in the phase III SPLENDOUR trial, we observed that both zoledronate and denosumab improved ICI efficacy with respect to ICI alone, with a sustained OS and an increased bone response rate evaluated by MD Anderson criteria. Conversely, consistent with data from clinical trials on ICIs, PFS in our patients was not improved (19, 37).

Another point to be explored is that bone response seems to be correlated to medical therapy due to the low rate of patients treated with radiotherapy.

Bearing in mind the caveat of the limited number of patients involved in our study, we nonetheless observed that denosumab worked rapidly, whereas zoledronate exerted its action after at least 6 months, which fits in with the known slow effect of this drug on bone homeostasis (38). These data strongly suggest that targeting the microenvironment to improve the efficacy of immunotherapy is a strategy worth considering. Another important indication comes from the NLR evaluated in our population. In patients with BMs from NSCLC receiving ICIs, an NLR cutoff ≤5 showed prognostic significance. Furthermore, this value changed in conjunction with a change in sensitivity to therapy, increasing in the event of disease progression or decreasing when response occurred (35).

Our study has a number of limitations, mainly that of limited sample size and the retrospective nature of the analysis (of note, the BMDA was prospectively built). Moreover, PD-L1 expression was not available for all patients. Despite these weaknesses, our data support the hypothesis that BTTs increase the activity of ICIs and reverse the negative impact of BMs on patient outcome. Larger prospective datasets or prospective randomized clinical trials are needed to provide more solid evidence of BTT potential.

There are still several open questions to be answered in the area of NSCLC, in particular how to overcome primary and acquired resistance to immunotherapy. This is often related to the status of the host’s immune homeostasis and involves myeloid-derived suppressor cells (MDSCs), tumor-associated macrophages, and T-regulatory cells, all of which play immune-suppressive roles. The use of zoledronate or denosumab in combination with ICIs could represent a potentially useful strategy to modulate the microenvironment and, consequently, the immune response.

## Conclusions

Our data suggest that BTT could potentially increase the efficacy of immunotherapy in NSCLC patients with BMs. Prospective trials are warranted to further investigate this finding.

## Data Availability Statement

The raw data supporting the conclusions of this article will be made available by the authors, without undue reservation.

## Ethics Statement

This study was reviewed and approved by the IRCCS IRST Ethics Committee (number of approval 1783/2014 of 20/03/2014). The patients/participants provided their written informed consent to participate in this study.

## Author Contributions

AB and TI conceived the idea for and designed the study. AB, JM, SS, FA, FR, VG, MRF, MB, and MF enrolled patients. FF performed the statistical analyses. VF, LM, and CS carried out the literature review. AB drafted the manuscript. All authors critically reviewed the manuscript, providing important feedback, and all read and approved the final version for submission.

## Conflict of Interest

The authors declare that the research was conducted in the absence of any commercial or financial relationships that could be construed as a potential conflict of interest.

## Publisher’s Note

All claims expressed in this article are solely those of the authors and do not necessarily represent those of their affiliated organizations, or those of the publisher, the editors and the reviewers. Any product that may be evaluated in this article, or claim that may be made by its manufacturer, is not guaranteed or endorsed by the publisher.

## Supplementary Material

Supplementary Figure 1 Differences between pre-treatment NLR values and NLR values **(A)** at disease progression (*p*-value from Wilcoxon signed-rank test: 0.027) and **(B)** at response (*p*-value from Wilcoxon signed-rank test: 0.030).
