# Supplementary figures and images for: Immune Checkpoint Inhibitors With or Without Bone-Targeted Therapy in NSCLC Patients With Bone Metastases and Prognostic Significance of Neutrophil-to-Lymphocyte Ratio
Source: Front Immunol. 2021 Nov 10;12:697298. doi: 10.3389/fimmu.2021.697298 (PMC8631508; doi:10.3389/fimmu.2021.697298)

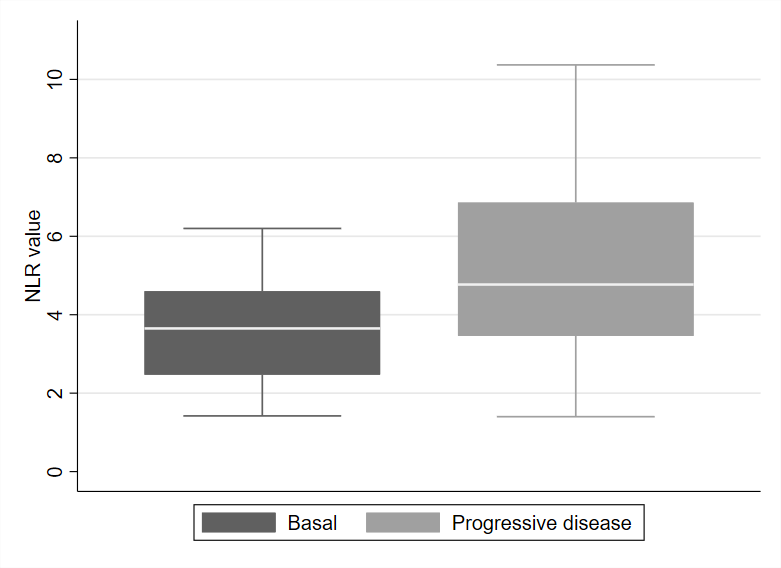

Supplement: Supplementary Figure 1 — Differences between pre-treatment NLR values and NLR values (A) at disease progression (p-value from Wilcoxon signed-rank test: 0.027) and (B) at response (p-value from Wilcoxon signed-rank test: 0.030). [file Image_1.tif]

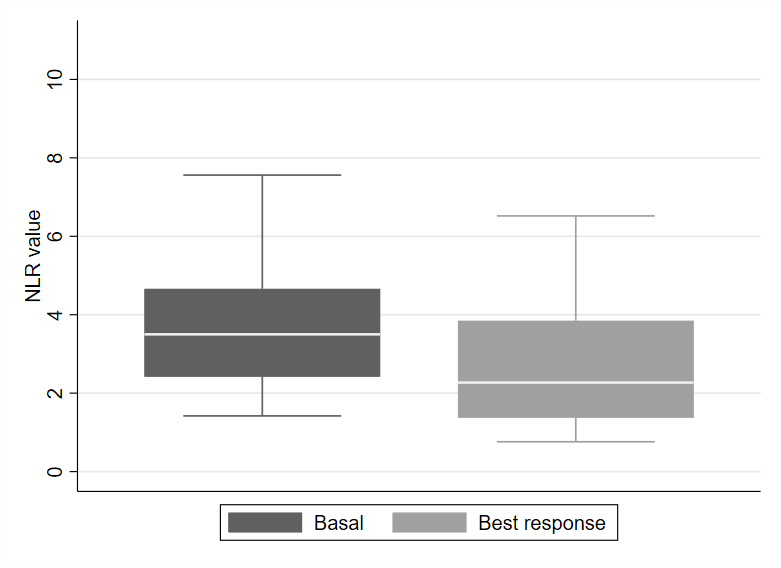

Supplement: Supplementary file 2 [file Image_2.tif]
