# Supplementary material for: Immune Checkpoint Inhibitors With or Without Bone-Targeted Therapy in NSCLC Patients With Bone Metastases and Prognostic Significance of Neutrophil-to-Lymphocyte Ratio
Source: Front Immunol. 2021 Nov 10;12:697298. doi: 10.3389/fimmu.2021.697298 (PMC8631508; doi:10.3389/fimmu.2021.697298)
Supplement: Supplementary file 3 [file Table_1.docx]

Supplementary tables

**Supplementary table 1. Patients’ characteristics according corresponding group.**

|  | **Only ICI** | **ICI + BTT** |
| --- | --- | --- |
| **Median age, years, at diagnosis of first bone metastasis (range)** | 65.5 (51-80) | 68.2 (53-80) |
|  | **No. (%)** | **No. (%)** |
| **Age (years) at diagnosis of primary bone metastasis** |  |  |
| ≤65 | 7 (43.8) | 11 (36.7) |
| >65 | 9 (56.2) | 19 (63.3) |
| **Gender** |  |  |
| Male | 14 (87.5) | 21 (70.0) |
| Female | 2 (12.5) | 9 (30.0) |
| **ECOG PS at diagnosis of first bone metastasis** |  |  |
| 0 | 6 (37.5) | 9 (30.0) |
| 1 | 10 (62.5) | 19 (63.3) |
| ≥2 | 0 (0.0) | 2 (6.7) |
| **Histology** |  |  |
| Adenocarcinoma | 14 (87.5) | 24 (80.0) |
| Squamous carcinoma | 2 (12.5) | 4 (13.4) |
| Adenosquamous carcinoma | 0 (0.0) | 1 (3.3) |
| Undifferentiated | 0 (0.0) | 1 (3.3) |
| **Grading (G)** |  |  |
| 1 | 1 (16.7) | 0 (0.0) |
| 2 | 2 (33.3) | 2 (18.2) |
| 3 | 3 (50.0) | 9 (81.8) |
| 4 | 0 (0.0) | 0 (0.0) |
| **Presence of visceral metastasis** |  |  |
| Yes | 13 (81.3) | 26 (86.7) |
| No | 3 (18.7) | 4 (13.3) |
| **Presence of brain metastasis** |  |  |
| Yes | 4 (25.0) | 9 (30.0) |
| No | 12 (70.0) | 21 (70.0) |
| **Presence of comorbidity** |  |  |
| Cardiovascular | 9 (56.3) | 13 (43.3) |
| Cardiovascular + other ^*^ | 2 (12.5) | 4 (13.3) |
| Other ^§^ | 1 (6.2) | 2 (6.7) |
| None | 4 (25.0) | 11 (36.7) |
| Line of therapy |  |  |
| First | 3 (18.8) | 7 (23.3) |
| Second | 9 (56.2) | 17 (56.7) |
| Third or more | 4 (25.0) | 6 (20.0) |

**Supplementary Table 2: Number and rate of patients who underwent Radiotherapy in the 2 subgroups.**

|  | **Only ICI**  **N° of pts (%)** | **ICI+Deno/Zol**  **N° of pts (%)** | **Total**  **N° of pts (%)** | **p-value**  **(chi square test)** |
| --- | --- | --- | --- | --- |
| **RT during ICI** |  |  |  |  |
| **No RT** | 13 (86.7) | 25 (89.3) | 38 (88.4) | 0.798 |
| **RT** | 2 (13.3) | 3 (10.6) | 5 (11.6) |  |
| **NE** | 1 | 2 | 3 |  |

RT: radiotherapy; NE: not evacuate

**Supplementary Table 3: Overall response according RECIST 1.1 criteria in patients treated with ICI alone or in association with BTT**

|  | **Only ICI**  **N° of pts (%)** | **ICI+Deno/Zol**  **N° of pts (%)** | **Total**  **N° of pts (%)** | **p-value**  **(chi square test)** |
| --- | --- | --- | --- | --- |
| **PR** | 1 (7.6) | 9 (64.6) | 10 (25.7) | 0.171 |
| **SD** | 6 (46.2) | 7 (26.9) | 13 (33.3) |  |
| **PD** | 6 (46.2) | 10 (38.5) | 16 (41.0) |  |
| **NV** | 3 | 4 | 7 |  |

**Supplementary Table 4: Overall response according MDA criteria in patients treated with ICI alone or in association with BTT**

|  | **Only ICI**  **N° of pts (%)** | **ICI+Deno/Zol**  **N° of pts (%)** | **Total**  **N° of pts (%)** | **p-value**  **(chi square test)** |
| --- | --- | --- | --- | --- |
| **Bone response** |  |  |  |  |
| **PR** | 2 (16.7) | 10 (43.5) | 12 (34.3) | 0.042 |
| **SD** | 7 (58.3) | 4 (17.4) | 11 (31.4) |  |
| **PD** | 3 (25.0) | 9 (39.1) | 12 (34.3) |  |
| **NE** | 4 | 7 | 11 |  |

NE: not evacuate ; PR: partial response; SD: stable disease; PD: progressive disease

**Supplementary Table 5: Multivariable models for Overall survival**

| Baseline covariates | Hazard Ratio (95%CI) | p-value |
| --- | --- | --- |
| **Treatment** |  |  |
| Only ICI vs ICI+BTT | 2.18 (0.6-7.6) | 0.222 |
| No ICI vs ICI+BTT | 6.06 (1.6-22.6) | 0.007 |
| **PS ECOG** |  |  |
| PS ECOG 1-3 vs PS ECOG 0 | 5.53 (1.7-18.3) | 0.005 |
| **K-RAS** |  |  |
| Mutated vs WT | 5.45 (1.7-17.6) | 0.005 |

**Supplementary table 6 on NLR prognostic value**

|  | **N° of pts** | **N° of events** | **Median OS (95%CI)** | **12-months OS (95%CI)** | **24-months OS (95%CI)** | **p-value log-rank test** |
| --- | --- | --- | --- | --- | --- | --- |
| **NLR before therapy** |  |  |  |  |  |  |
| ≤5 | 26 | 13 | 21.8 (15.4-NE) | 80.4 (59.2-91.4) | 47.6 (27.3-65.5) | 0.042 |
| >5 | 10 | 8 | 14.5 (5.6-32.9) | 67.5 (29.1-88.3) | 33.8 (7.9-62.7) |  |
